# The Histone Deacetylase Inhibitors MS-275 and SAHA Suppress the p38 Mitogen-Activated Protein Kinase Signaling Pathway and Chemotaxis in Rheumatoid Arthritic Synovial Fibroblastic E11 Cells

**DOI:** 10.3390/molecules181114085

**Published:** 2013-11-14

**Authors:** Qiu-Yi Choo, Paul C Ho, Yoshiya Tanaka, Hai-Shu Lin

**Affiliations:** 1 Department of Pharmacy, Faculty of Science, National University of Singapore, 10 Kent Ridge Crescent, 119260, Singapore; E-Mails: choo_qiuyi@hsa.gov.sg (Q.-Y.C.); paul.ho@nus.edu.sg (P.C.H.); 2 First Department of Internal Medicine, School of Medicine, University of Occupational and Environmental Health, 1-1 Iseigaoka, Yahatanishi-ku, Kitakyushu 807-8555, Japan; E-Mail: tanaka@med.uoeh-u.ac.jp

**Keywords:** histone deacetylase inhibitor, rheumatoid arthritis, MS-275, SAHA, p38 MAPK, MKP-1, chemotaxis

## Abstract

MS-275 (entinostat) and SAHA (vorinostat), two histone deacetylase (HDAC) inhibitors currently in oncological trials, have displayed potent anti-rheumatic activities in rodent models of rheumatoid arthritis (RA). To further elucidate their anti-inflammatory mechanisms, the impact of MS-275 and SAHA on the p38 mitogen-activated protein kinase (MAPK) signaling pathway and chemotaxis was assessed in human rheumatoid arthritic synovial fibroblastic E11 cells. MS-275 and SAHA significantly suppressed the expression of p38α MAPK, but induced the expression of MAPK phosphatase-1 (MKP-1), an endogenous suppressor of p38α in E11 cells. At the same time, the association between p38α and MKP-1 was up-regulated and consequently, the activation (phosphorylation) of p38α was inhibited. Moreover, MS-275 and SAHA suppressed granulocyte chemotactic protein-2 (GCP-2), monocyte chemotactic protein-2 (MCP-2) and macrophage migration inhibitory factor (MIF) in E11 cells in a concentration-dependent manner. Subsequently, E11-driven migration of THP-1 and U937 monocytes was inhibited. In summary, suppression of the p38 MAPK signaling pathway and chemotaxis appear to be important anti-rheumatic mechanisms of action of these HDAC inhibitors.

## 1. Introduction

Histone deacetylase (HDAC) inhibitors are a new class of anti-cancer agents that have attracted significant interest in drug discovery during the past 10-15 years [1]. SAHA (vorinostat) and FK228 (romidepsin) been approved by the US Food and Drug Administration for the treatment of relapsed cutaneous T-cell lymphoma [1,2]. The efficacy of SAHA, FK228 and several HDAC inhibitors in other oncological conditions are under extensive clinical investigation [1,2]. Besides cancer, HDAC inhibitors also displayed therapeutic potential in neurodegeneration, neuromuscular and cardiac diseases, and a variety of autoimmune disorders such as inflammatory bowel diseases and multiple sclerosis as well as systemic lupus erythematosus [1,2,3,4,5].

MS-275 and SAHA are two structurally distinct orally active histone deacetylase (HDAC) inhibitors currently being studied in various oncological trials [1,2]. Their *in vivo* anti-inflammatory activities have been observed in pre-clinical models of rheumatoid arthritis (RA) [6]. As a prophylactic agent, MS-275 almost blocked the onset of collagen-induced arthritis in mice [6]. Moreover, therapeutic intervention with MS-275 suspended the disease progression and joint destruction in rats [6]. The disease-modifying activities of MS-275 appeared to be stronger than those of methotrexate, which is a first-line anti-rheumatic agent [6]. Similarly, SAHA also possessed moderate preventive efficacy in both mice and rats [6]. The molecular anti-rheumatic mechanisms of MS-275 and SAHA are being elucidated.

Tumor necrosis factor-α (TNF-α), interleukin-1 (IL-1) and interleukin-6 (IL-6) are crucial pro-inflammatory cytokines that drive joint inflammation in RA [7,8]. Blockade of such cytokines offers clinical therapeutic strategies for treating RA [7,8]. The transcriptional factor nuclear factor-κB (NF-κB) plays an important role in the pathogenesis of RA as NF-κB activation up-regulates these pro-inflammatory cytokines [9,10]. In a recent study, we identified suppression of NF-κB signaling as one of the major anti-rheumatic mechanisms of MS-275 and SAHA [11], but other signaling pathways may also contribute to the anti-rheumatic activities of HDAC inhibitors.

The p38 mitogen-activated protein kinase (MAPK) signaling pathway is involved in many physiological processes [12,13]. This pathway is crucial for the induction and maintenance of chronic inflammation [14]. Like NF-κB, the p38 MAPK signaling pathway has been implicated as a key regulator in the production of RA driving pro-inflammatory cytokines and downstream signaling events leading to joint inflammation and destruction [15]. Therefore, the p38 MAPK pathway has emerged as an interesting molecular target of small molecule inhibitors for RA therapy [15,16], although their effects on the p38 MAPK signaling pathway remain unclear.

The onset of arthritis involves the infiltration of inflammatory cells to RA-affected joints [8,17]. Chemotaxis, which is mediated by various chemotactic factors including cytokines and chemokines facilitates this process [8,17]. In our previous study, we observed that MS-275 and SAHA suppressed various inflammatory mediators such as nitric oxide (NO), IL-1β, IL-6, IL-18 and TNF-α [11] and as a result RA-related chemotaxis may also be inhibited. In this study, we assessed the impact of MS-275 and SAHA on the p38 MAPK signaling pathway and chemotaxis in RA synovial fibroblastic E11 cells. Our study provides useful information to further elucidate the anti-rheumatic mechanisms of HDAC inhibitors.

## 2. Results and Discussion

### 2.1. MS-275 and SAHA Suppressed p38 MAPK Signaling Pathway

**Figure 1:**
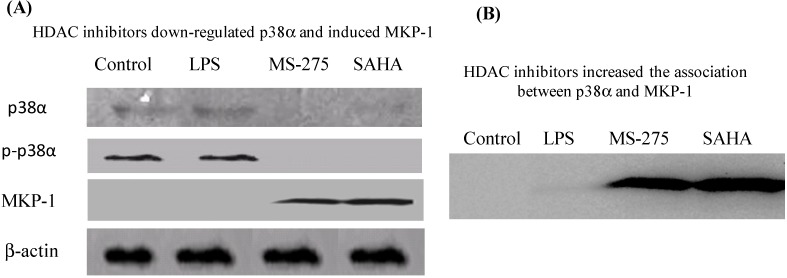
HDAC inhibitors suppressed p38 MAKP signal pathway. (**A**) MS-275 and SAHA down-regulated p38α, phosphorylated-p38α (p-p38α) and induced MKP-1. E11 cells were incubated with HDAC inhibitors (50 nM) for 1 h before they were stimulated by LPS. 24 h later, the profiles of p38α, p-p38α and MKP-1 were assessed by western blotting. Gel sections were obtained from different gels for each tested antibody, as indicated by the divided lines. (**B**) MS-275 and SAHA enhanced association between p38 and MKP-1. E11 cells were incubated with HDAC inhibitors (50 nM) for 1 h before they were stimulated by LPS. 24 h later, the nuclear extract was immunoprecipitated with antibody specific to MKP-1. p38α associated with MKP-1 was visualized by western blotting.

The impact of MS-275 and SAHA on p38α and MAPK phosphatase-1 (MKP-1) were assessed by western blot (Figure 1A). p38α was expressed and activated (phosphorylated) in E11 cells even without LPS stimulation. This was in agreement with the clinical observations [8,14]. LPS did not alter p38α or p-p38α protein levels. However, MS-275 and SAHA (50 nM) dramatically suppressed p38α expression and activation. The activity of p38 MAPK is tightly regulated by MKP-1, an endogenous inhibitor for p38α [14]. MKP-1 was not detected in non-treated or LPS-stimulated E11 cells. On the other hand, MS-275 and SAHA significantly induced MKP-1 (Figure 1A). Furthermore, the association between p38α and MKP-1 was enhanced after HDAC inhibitor treatment (Figure 1B). The suppressive effects of MS-275 and SAHA on p38 MAKP signaling appeared to be due to the combined effects of inhibition of p38α and induction of MKP-1 expression, which deactivates p38α by the removal of the phosphate at the 180 and 182 amino acid residues [14].

### 2.2. MS-275 and SAHA Suppressed Secretion of Granulocyte Chemotactic Protein-2 (GCP-2), Monocyte Chemotactic Protein-2 (MCP-2) and Macrophage Migration Inhibitory Factor (MIF) in E11 cells

**Figure 2:**
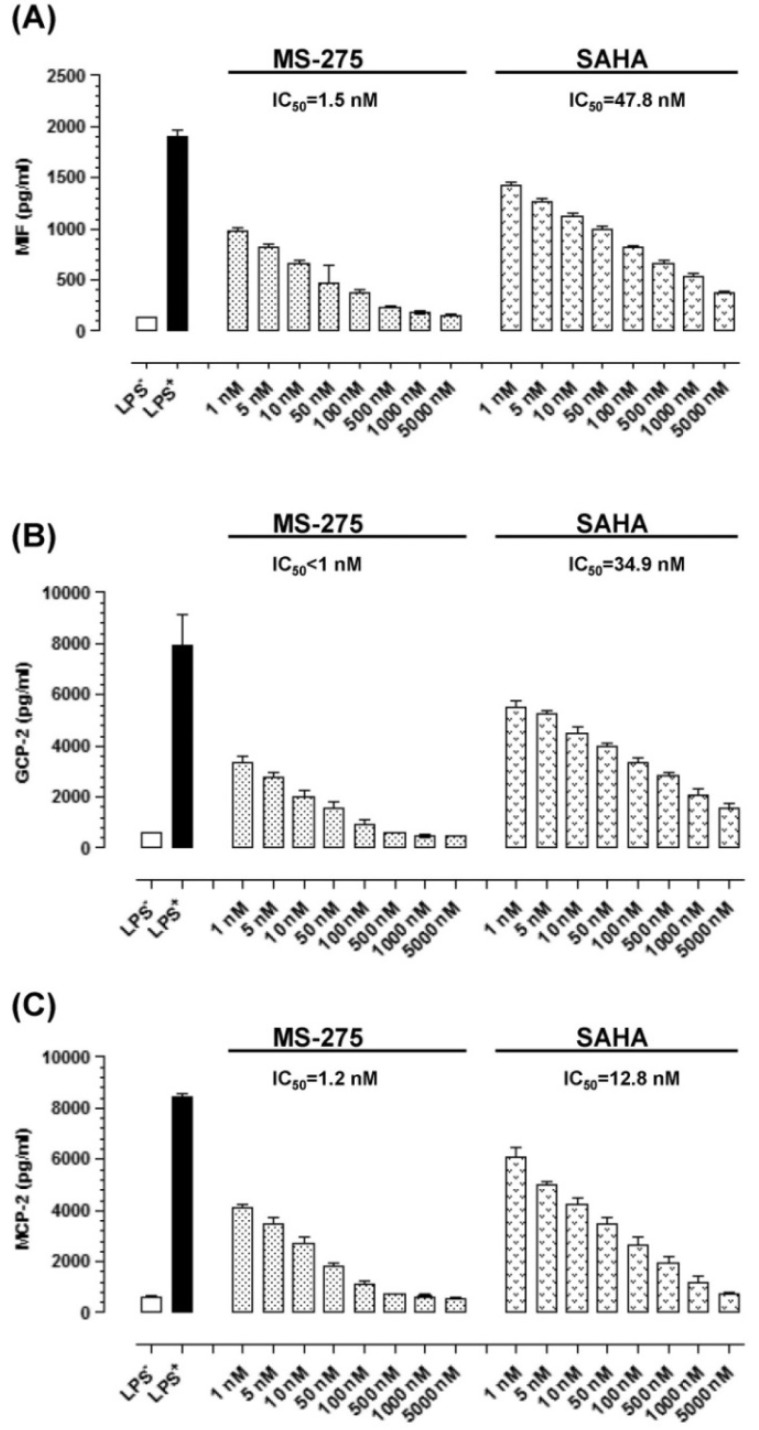
MS-275 and SAHA suppressed the secretion of GCP-2, MCP-2 and MIF in E11 cells. (**A**) GCP suppression. (**B**) MCP-2 suppression. (**C**) MIF suppression. E11 cells were incubated with HDAC inhibitors at various concentrations for 1 h before they were stimulated by LPS. 24 h later, the secretion of GCP-2, MCP-2 and MIF were quantified by ELISA (n = 8). Bars show the mean and SD in (**A**–**C**).

The impact of MS-275 and SAHA on the secretion of GCP-2, MCP-2 and MIF was assessed by ELISA (Figure 2). E11 possesses low level baseline GCP-2, MCP-2 and MIF secretion. LPS enhanced their secretion by about 10-fold. MS-275 and SAHA suppressed the secretion of these three signal molecules in a concentration-dependent manner. Interestingly, the IC_50_ levels of MS-275 were less than 2 nM and those of SAHA were less than 50 nM. These IC_50_ levels were comparable to the IC_50_ levels required to inhibit proliferation, NF-κB activation, and NO, IL-18 as well as VEGF secretion in E11 cells [11].

### 2.3. MS-275 and SAHA Inhibited E11-Driven Monocyte Migration

**Figure 3:**
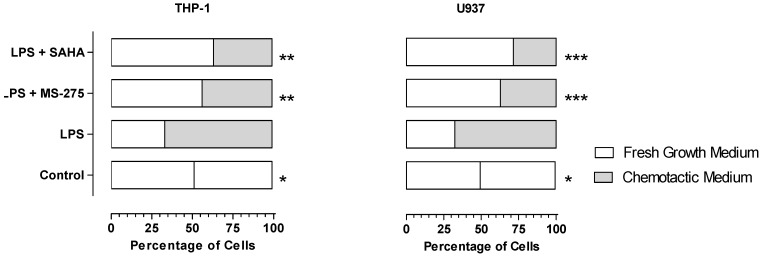
MS-275 and SAHA inhibited E11-driven migration of THP-1 and U937 cells. Number of cells migrated into fresh growth medium and chemotactic medium (in which E11 cells were grown in the presence or absence of LPS (5 μg/mL) and HDAC inhibitors (100 nM) for 24 h) were counted under a light microscope. The distribution profiles for the control and various treatment groups were expressed in percentages and depicted in a bar chart. * = *p* < 0.05, ** = *p* < 0.01, *** = *p* <0.001 (between this group and LPS group, two-tailed Chi-square test).

The results of E11-driven monocyte migration are summarized in Figure 3. Under control conditions, THP-1 and U937 cells distributed equally into fresh medium in the wells. LPS could stimulate E11 cells to secrete various chemotactic factors. As a result, about two thirds of THP-1 and U937 cells distributed into chemotactic medium. In the presence of HDAC inhibitor, the distribution trend was reversed and more than 50% of the cells distributed into fresh medium. Clearly, MS-275 and SAHA inhibited E11-driven migration of THP-1 and U937 cells.

RA synovial fibroblasts (RASFs) are key players in RA pathogenesis and joint destruction [18,19]. They appear to be distinct from synovial fibroblasts isolated from osteoarthritis or non-arthritis patients. RASFs possess tumor-like characteristics such as anchorage-independent growth, loss of contact inhibition, oncogene activation, monoclonal or oligoclonal expansion, detectable telomerase activity and somatic gene mutations [19]. Recent studies even showed that RASFs spread RA to unaffected joints [20]. As RASFs are crucial in RA pathogenesis, this study was also focused on RASF-like E11 cells.

The p38 MAPK kinase family consists of α, β, γ and δ isoforms [14,21]. p38α appears to be most relevant to RA as it is highly expressed and activated in RA synovium and regulates inflammation through the production of TNF-α, IL-1 and IL-6 [14,21]. Moreover, p38α had been proposed as a target for RA therapy and small molecule inhibitors have been developed and tested clinically [14,21], therefore, in this study, we assessed the impact of HDAC inhibitors MS-275 and SAHA on p38α in E11 cells.

MS-275 and SAHA were found to suppress p38 MAPK signaling pathway by two apparently distinct mechanisms, namely down-regulation of p38α expression and induction of MKP-1, a well known negative regulator of p38 MAPK. The induction of MKP-1 by FK-228, a cyclic peptide HDAC inhibitor, had been reported in some cancer cells [22]. In addition, the activities of MKP-1 might also be up-regulated by HDAC inhibitors. Cao *et al*. reported that MKP-1 was acetylated by p300 on lysine residue K57 within its substrate-binding domain [23]. The acetylation of MKP-1 enhanced its interaction with p38, thereby increasing its phosphatase activity and interrupting p38 MAPK signaling [23]. Therein, HDAC inhibitor trichostatin A increased MKP-1 acetylation and blocked p38 MAPK signaling [23].

To our knowledge, this is the first study on the impact of HDAC inhibitors on p38 MAPK signaling pathway in RASFs. It would be of interest to confirm such findings in an *in vivo* model of RA in future studies. The suppressive effects on p38 by other HDAC inhibitors have been observed in some inflammation-related *in vitro* models [24,25,26]. Interestingly, the anti-cancer activities of HDAC inhibitors were usually associated with p38 activation [27,28,29]. Clearly, the effects of HDAC inhibitors on the p38 signaling pathway depend on the cell and the stimuli types [24,25,26,27,28,29,30].

Although p38 has been considered as a target for RA treatment [14], small molecule p38α inhibitors did not display good clinical efficacy in trials [21]. Possible explanations have been proposed: as the inflammatory signal networks of RA are highly redundant and complex, blockade of a downstream pathway may be insufficient to fight RA [21], and therefore, it may be more practical to work on upstream pathway(s) or decrease the selectivity of inhibitors [21]. Our results support such a hypothesis. Aside from suppressing the p38 MAPK pathway, HDAC inhibitors also induced p21 (a cyclin-dependent kinase inhibitor which possesses anti-proliferative/anti-invasive activities) and inhibited the NF-κB signaling pathway [11] (a pivotal switch in inflammation) [10]. Clearly, HDAC inhibitors display anti-rheumatic activities via multiple mechanisms and may be more successful in counteracting the highly redundant inflammatory network.

MIF is a pivotal regulator of innate immunity [31]. Its pro-inflammatory role in RA pathogenesis had been established and blockade of MIF by monoclonal antibody could offer *in vivo* anti-rheumatic efficacy [31]. Hence, MIF appears to be a target for RA therapy [31]. In this study, potent suppressive effects on MIF were observed with MS-275 and SAHA. To our knowledge, this is the first report of the suppressive effect of HDAC inhibitors on MIF in RASFs. As MIF is an activator of p38 [31], inhibition of MIF may further attenuate p38 MAPK signaling pathway. Recent evidence indicated that MIF played an important role in atherosclerosis [31]. Suppression of MIF by HDAC inhibitors may offer extra benefits for RA patients, who have a higher incidence of atherosclerosis [31].

The recruitment of leukocytes and lymphocytes from the blood stream into the inflamed joint plays an important role in RA pathogenesis [8]. Chemokines are crucial for such processes [8]. In this study, MS-275 and SAHA inhibited chemokines GCP-2 and MCP-2 and hence, chemotaxis in E11 cells. Prior to these observations, the inhibition of chemokines GCP-2 and MCP-2 by HDAC inhibitors has never been attempted. The anti-chemotatic activities of MS-275 and SAHA might be attributed to the suppression of inflammatory mediators such as cytokines [11], chemokines (presented in the current study) and nitric oxide [11]. As the expression of these inflammatory mediators are controlled by the NF-κB and p38 MAPK signaling pathways [8], the suppressive effects of HDAC inhibitors on these two pathways may be able to inhibit RA-related chemotasis.

The pharmacokinetic profile of MS-275 and SAHA had been reported from the clinical trials [32,33]. According to our *in vitro* studies, the effective concentrations for MS-275 and SAHA to display anti-rheumatic activities were usually less than 100 nM. Clearly, such concentrations were much lower than the levels required to exhibit anti-cancer effects and could be achieved and maintained for a prolonged duration after oral administration. Therefore, there lies the possibility to use MS-275 and SAHA as innovative disease-modifying anti-rheumatic agents.

Although both MS-275 and SAHA are HDAC inhibitors, their inhibitory specificity against individual HDAC isoforms is different [34]. Besides the current study, MS-275 was also found to possess stronger anti-rheumatic activities than SAHA in two previous studies [6,11]. It is still unclear whether the anti-rheumatic effects of HDAD inhibitors are mediated by the inhibition of some specific HDAC isoform(s). Therefore, it is of great interest to identify the HDAC isoform(s) to which the anti-rheumatic activities may be attributed in future studies. Isoform-specific HDAC inhibitors may be more favorable as they may minimize the side effects/toxicities.

## 3. Experimental

### 3.1. HDAC Inhibitors

MS-275 (purity > 95%) and SAHA (purity > 98%) were supplied from Axxora (San Diego, CA, USA) and Toronto Research Chemicals Inc. (North York, ON, Canada), respectively. 

### 3.2. Cell Culture

E11 is a cell line established from human RA synovial fibroblast [35]. Human monocytic THP-1 and U937 cell lines were purchased from American Type Culture Collection (Manassas, VA, USA). E11 and U937 cells were cultured in Roswell Park Memorial Institute (RPMI) 1640 medium (Invitrogen, Carlsbad, CA, USA) in the presence of 10% (v/v) heat-inactivated fetal bovine serum (FBS) (HyClone, Waltham, MA, USA). THP-1 cells were also cultured in RPMI 1640 medium supplemented with 20% FBS and 0.05 nM 2-mercaptoethanol (Sigma-Aldrich, St. Louis, MO, USA). 1% (v/v) antibiotic-antimycotic solution (Invitrogen) was spiked into all culture media to prevent contamination. All cell cultures were maintained at 37 °C in a humidified atmosphere with 5% CO_2_. Such cell culture protocols have also been applied in our recent study [11].

### 3.3. Western Blotting Analyses of p38α, Phosphate-p38α (p-p38α) and MAPK Phosphatase (MKP)-1

E11 cells were seeded in 6 cm petri dishes and incubated for 24 h before HDAC inhibitors were added (final concentration 50 nM). After 1 h, lipopolysaccharide (LPS, final concentration 5 μg/mL, Sigma-Aldrich) was added. After 24 h, the medium was aspirated and cells were washed with ice-cold PBS before being scraped off the culture dish. The cells were treated with 100 mL of lysis buffer (1% Triton X-10, protease inhibitor and 50 mM Tris–HCl pH 7.4). After a 30 min ice bath, the samples were centrifuged at 1100 g for 10 min at 4 °C. Separation of proteins in the supernatant were achieved by sodium dodecyl sulfate polyacrylamide gel electrophoresis (SDS-PAGE) using a (×2) SDS loading buffer (0.02% bromophenol blue, 0.2M dithiothreitol (DTT), 20% glycerol, 8% SDS and 0.25M Tris-HCl pH 6.8). The proteins loaded on SDS-PAGE were equalized using bicinchoninic acid (BCA) assay (Pierce Biotechnology, Rockford, IL, USA) and resolved by a 12.5% SDS-PAGE gel at 150 V for 45 min.

After electrophoresis, separated proteins were transferred to polyvinylidene fluoride membranes in a transfer buffer (192 mM glycine, 20% methanol and 25 mM Tris-HCl pH 8) at a constant voltage of 125 V for 1 h at 4 °C in an electro-transfer unit (Bio-Rad Laboratories, Hercules, CA, USA). The membranes were then incubated in 2% (w/v) solution of non-fat milk dissolved in PBS (MPBS) for 1 h at room temperature. They were probed with primary antibodies against p38α, p-p38α, MKP-1 and β-actin (p38α: sc-535, p-p38α: sc-17852R, MKP-1: sc-370; Santa Cruz Biotechnology, Inc. (Santa Cruz, CA, USA)), which were diluted in 3% BSA. The membrane was kept at 4 °C overnight. On the second day, the membrane was washed thrice with PBS before incubation for 1 h at room temperature with secondary antibodies (peroxidase conjugated ImmunoPure goat anti-mouse IgG or goat anti-rabbit IgG; Pierce Biotechnology) diluted in 2% (w/v) MPBS. Subsequently, the membrane was washed thrice with PBS. SuperSignal West Pico Chemiluminescent Substrate (Pierce Biotechnology) was added to the membrane and the resultant signals were acquired by a MultImage Light Cabinet (Alpha Innotech, San Leandro, CA, USA). This western blotting protocol has also been used in our recent study [11].

### 3.4. Association of MKP-1 with p38α

E11 cells were treated with HDAC inhibitors and LPS as described above. The nuclear fraction was extracted by a universal magnetic co-immunoprecipitation (Co-IP) kit (Active Motif, Carlsbad, CA, USA) and immunoprecipitated with antibody to MKP-1 (sc-370). The immunoprecipitants were immunoblotted with antibody to p38α (sc-535). The results were visualized with a MultImage Light Cabinet.

### 3.5. Secretion of GCP-2, MCP-2 and MIF

Human granulocyte chemotactic protein-2 (GCP-2 or CXCL6) and monocyte chemotactic protein-2 (MCP-2 or CCL8) ELISA sets were purchased from Antigenix America Inc. (Huntington, NY, USA) while that for macrophage migration inhibitory factor (MIF) was purchased from R & D Systems (Minneapolis, MN, USA). E11 cells were seeded in 96-well plates and incubated for 24 h before HDAC inhibitors (final concentration 1–5,000 nM) were added. After 1 h, LPS (final concentration 5 μg/mL) was added and incubated for another 24 h. GCP-1, MCP-2 and MIF secreted into the supernatant were quantified by ELISA (*n* = 4).

### 3.6. Chemotaxis Assay

In order to determine if HDAC inhibitors influenced monocyte chemotaxis, assays were performed based on a well-established protocol [36]. A series of three wells (2.4 mm in diameter and 2.4 mm apart) were cut in a 6 cm petri dish containing agarose gel 2.4% (w/v). The center well of each thee well series received 10 μL of THP-1 or U937 cell suspension (2 × 10^5^ cells/mL). One well received 10 μL of chemotatic medium, in which E11 cells were grown in the presence or absence of LPS (5 μg/mL) and HDAC inhibitors (100 nM) for 24 h. The remainder well received 10 μL of fresh growth medium. Completed dishes were incubated at 37 °C for 24 h. Cells were fixed by adding absolute methanol followed by 37% formaldehyde for 30 min each. The plates were then stained with Field’s stain (Sigma-Aldrich) and air dried. The number of cells that migrated to the chemotactic and fresh medium was counted under a light microscope.

### 3.7. Data Analysis and Statistics

All the 50% inhibitory concentration (IC_50_) values and statistical analyses were performed using GraphPad Prism 5.03 (GraphPad Software, Inc., La Jolla, CA, USA). Two-tailed t-test was used to compare the chemokine data. Chemotaxis data was analyzed with Chi-square test. *P* values less than 0.05 were considered to be statistically significant.

## 4. Conclusions

In summary, MS-275 and SAHA down-regulated p38α and induced MKP-1 in E11 cells. The secretions of GCP-2, MCP-2 and MIF as well as E11-driven monocyte migration were also inhibited. Suppression of the p38 MAPK signaling pathway and chemotaxis appear to be important anti-rheumatic mechanisms of these HDAC inhibitors.
